# The complete chloroplast genome sequence of *Carpinus tibetana* (Betulaceae)

**DOI:** 10.1080/23802359.2020.1851151

**Published:** 2021-01-17

**Authors:** Renping Xu, Guili Wu

**Affiliations:** State Key Laboratory of Grassland Agro-Ecosystem, Institute of Innovation Ecology & School of Life Sciences, Lanzhou University, Lanzhou, China

**Keywords:** Carpinus tibetana, chloroplast genome, phylogenetic analysis

## Abstract

The complete chloroplast genome of *Carpinus tibetana* was a circular DNA molecule of 158,762 bp in length, containing a large single copy region (LSC) of 87,825 bp and small single copy region (SSC) of 18,797 bp, which were separated by a pair of 26,071 bp and 26,069 bp inverted repeat regions (IRs). The all GC content of *C. tibetana* chloroplast genome was 36.47%. It encoded totally 127 genes, including 83 protein-coding genes, 35 tRNA genes and eight rRNA genes. The chloroplast genome of *C. tibetana* will provide useful genetic information for future conservation genetics and phylogenetic studies.

*Carpinus tibetana*, which belongs to the birch family Betulaceae, is an endemic species to China. The common name of *Carpinus* species is hornbeam, which deriving from the hardness of the woods. Hornbeams yield a very hard timber and usually used to construct carving boards, tool handles, handplane soles and other products where a very tough, hard wood is required (Eichhorn and Haran 2012). There are 30–40 species occurred across much of the temperate regions of the northern hemisphere, with the greatest number of species in east Asia, particularly China (Li and Skvortsov 1999). *Carpinus tibetana* is a new published species and narrowly distributed at 1550–2300 m a.s.l. regions in Bomi and Motuo Counties, Xizang Autonomous Region (Tibet), China (Lu et al. 2018). As the rare high altitude tree species, small population size and the considerable economic values, the genomic information is urgently need to help utilizing and protecting the resource of *C. tibetana*.

Fresh leaves of *C. tibetana* were collected from Tongmai, Bomi, Tibet, China (95°05′E, 30°06′N, 2060 m). The voucher specimen was deposited at Lanzhou University under the accession number of lzu-2019-Cti20. Total DNA was used to generate libraries with insert size of 500 bp, and a total of 4.7 Gb raw reads were generated by Illumina Hiseq 2000 Platform. The chloroplast genome was assembled by NOVOPlasty (Dierckxsens et al. 2017) with *matK* gene from *C. cordata* as the seed (GenBank accession no. NC036723), and genome annotation was performed by Plann (Plastome Annotator) (Huang and Cronk 2015) and Geneious (Kearse et al. 2012). The chloroplast genome was submitted to GenBank (accession no. MT727002).

The complete chloroplast genome of *C. tibetana* was a circular DNA molecule of 158,762 bp in length, which contains two reverse repeated regions (IRs) of 26,071 bp and 26,069 bp each separated by a large single-copy region (LSC) and a small single-copy region (SSC) of 87,825 bp and 18,797 bp, respectively. The chloroplast genome encoded a set of 127 genes, containing 83 protein-coding genes, 35 tRNA genes and eight rRNA genes. GC content of the whole genome, IRs, LSC and SSC regions are 36.47%, 42.45%, 34.29% and 30.04%, respectively. Seventeen genes contained introns, fourteen of which contained one intron while three genes (*clpP*, *ycf3* and *rps12*) contained two introns.

To investigate the phylogenetic position of *C. tibetana*, the chloroplast genome sequences of 16 Betulaceae species plus one Fagaceae species (*Quercus aquifolioides*) were selected to construct phylogenetic tree. The sequences were aligned by MAFFT version 7 (Katoh and Standley 2013). The maximum likelihood (ML) tree was inferred using RaxML version 8 (Stamatakis 2014) with 1000 bootstraps. The results indicated that four *Carpinus* species were clustered together, and *Carpinus* and *Ostrya* had a close relationship (Figure 1). Our chloroplast genome of *C. tibetana* will provide useful genetic information for further studies associated with speciation, adaptation and conservation of *Carpinus* species.

**Figure 1.**
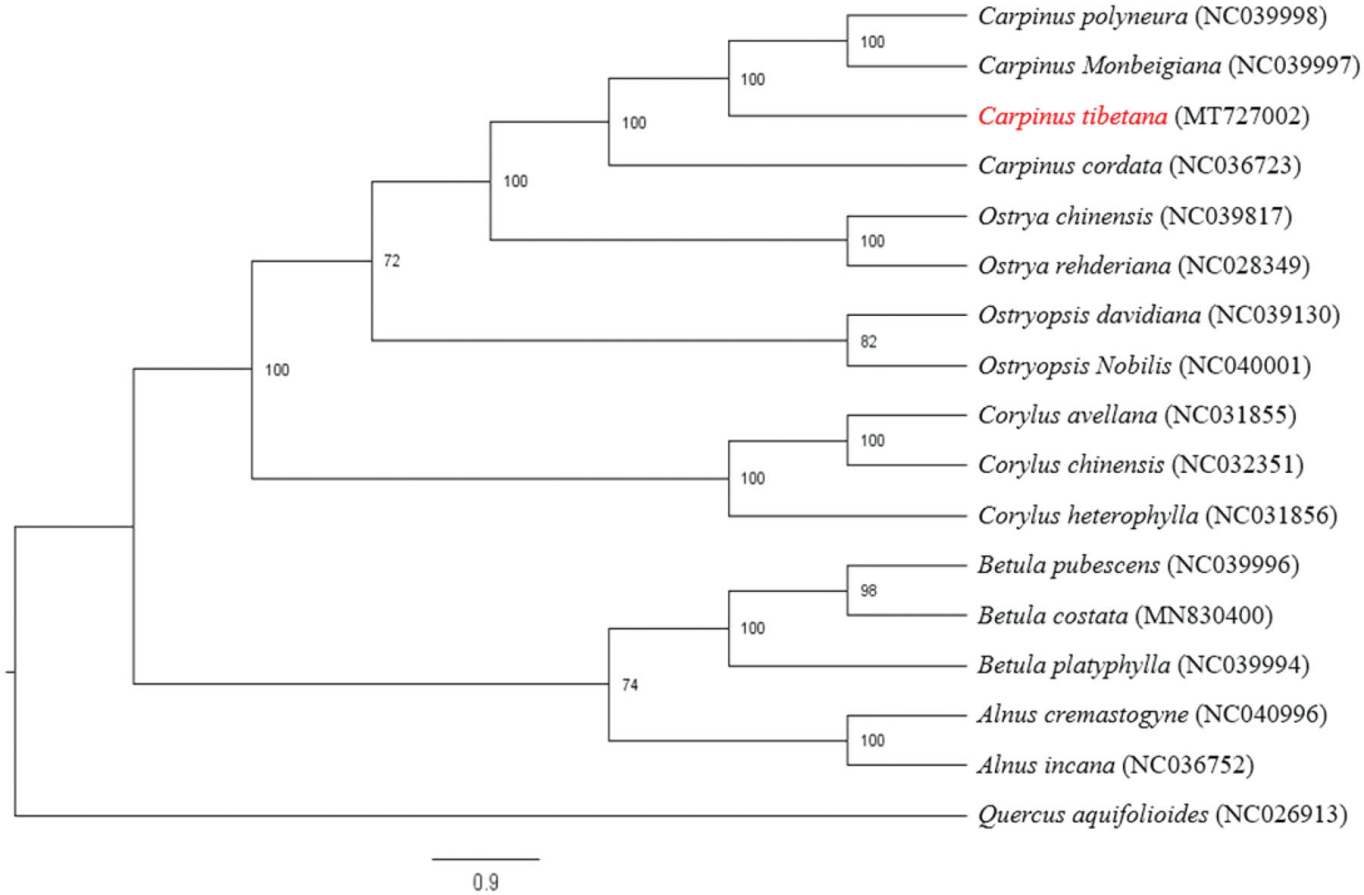
Phylogenetic relationships among 17 complete chloroplast genomes. Bootstrap support values are given at the noes. Chloroplast genome accession number used in this phylogeny analyses: *Carpinus polyneura*: NC039998; *Carpinus monbeigiana*: NC039997; *Carpinus tibetana*: MT727002 (the sample in this study); *Carpinus cordata*: NC036723; *Ostrya chinensis*: NC039817; *Ostrya rehderiana*: NC 028349; *Ostryopsis davidiana*: NC039130; *Ostryopsis nobilis*: NC040001; *Corylus avellana*: NC031855; *Corylus chinensis*: NC032351; *Corylus heterophylla*: NC031856; *Betula pubescens*: NC039996; *Betula costata*: MN830400; *Betula platyphylla*: NC039994; *Alnus cremastogyne*: NC040996; *Alnus incana*: NC036752; *Quercus aquifolioides*: NC026913.

## Data Availability

The data that support the findings of this study are openly available in GenBank of NCBI at http://www.ncbi.nlm.nih.gov, reference number MT727002, SRA accession number SRR12695871.
